# Effect of the Composition of Polymer-Composite Lightweight Concrete for Floating Platforms on Their Thermal and Strength Properties

**DOI:** 10.3390/polym18121518

**Published:** 2026-06-18

**Authors:** Alexey N. Beskopylny, Sergey A. Stel’makh, Evgenii M. Shcherban’, Diana M. Shakhalieva, Andrei Chernil’nik, Ivan Panfilov, Nikita Beskopylny, Yasin Onuralp Özkılıç

**Affiliations:** 1 Department of Transport Systems, Faculty of Roads and Transport Systems, Don State Technical University, Rostov-on-Don 344003, Russia; nbeskopylnyi@donstu.ru; 2 Department of Unique Buildings and Construction Engineering, Don State Technical University, Rostov-on-Don 344003, Russia; sstelmah@donstu.ru (S.A.S.); achernilnik@donstu.ru (A.C.); 3 Department of Engineering Geometry and Computer Graphics, Don State Technical University, Rostov-on-Don 344003, Russia; etsherban@donstu.ru; 4 Department of Design, Don State Technical University, Rostov-on-Don 344003, Russia; delshaeva@donstu.ru; 5 Department of Theoretical and Applied Mechanics, Agribusiness Faculty, Don State Technical University, Gagarin Square, Rostov-on-Don 344003, Russia; panfilov.i@gs.donstu.ru; 6 Department of Civil Engineering, Faculty of Engineering, Necmettin Erbakan University, Konya 42000, Turkey; 7 Department of Technical Sciences, Western Caspian University, Baku 1001, Azerbaijan

**Keywords:** lightweight concrete, polymer-composite, polypropylene, polyamide, polyester, thermal properties, compression strength

## Abstract

In recent years, the incorporation of voids with different geometrical configurations has emerged as one of the most effective strategies for reducing concrete consumption in structural systems. This article is devoted to the numerical analysis and experimental study of lightweight concrete using polymer spheres of various diameters and properties as voids. Lightweight concrete specimens with polypropylene spheres of 10 mm, 12 mm, 15 mm, 19.05 mm, and 20 mm diameters were manufactured. The experimental specimens were subjected to compression tests, and the results were compared with the numerical model. A numerical model employing the Menetrey–Willam constitutive model was established using spheres with comparable diameters and various types of polymers: polypropylene, polyamide 66, and polyester. The thermal properties of polymer-composite lightweight concrete (PCLC) were determined for various wall thicknesses using different polymers. The results demonstrated a 1% to 2% lower thermal conductivity coefficient for PCLC with polypropylene compared to polyethylene. Verification of the compressive strength results by comparing the data with the experiment demonstrated good accuracy in predicting the strength and deformation properties. The calculated stress and strain field distributions enabled the identification of the cracking patterns and failure mechanisms of the specimens containing polymer spheres. It has been proven that smaller radius spheres manufactured from higher modulus polymer materials have better deformation resistance and provide good, consistent strength in lightweight polymer concrete. The observed strength reduction in PCLC regarding the control composition (*R_b_* = 21.4 MPa) without spheres is within 11% for 10 mm (*R_b_* = 19.1 MPa) and 12 mm spheres (18.2 MPa). For larger-diameter spheres, the strength reduction reaches 25% (*R_b_* = 16 MPa).

## 1. Introduction

In recent years, the demand for innovative floating platform construction technology has grown due to the expansion of various industries requiring work in marine and aquatic environments [1]. Key factors influencing the development of new technologies and materials for floating platforms include energy projects [2,3], the development of renewable energy [4], the construction and modernization of marine and coastal infrastructure, and other factors. There is a growing need for adaptable and cost-effective solutions for the maintenance, installation, and inspection of facilities in complex aquatic environments. Floating platforms are used in the oil and gas industry, particularly for drilling, maintenance, and hydrocarbon production. They are also used in renewable energy projects—for the construction, maintenance, and operation of offshore wind farms [5,6]. The development of innovative technologies has contributed to the emergence of lightweight concrete structures reinforced with polymer fibers [7]. The global shift toward sustainable energy solutions is driving demand for floating platforms, as they enable the installation of wind turbines in locations where traditional installations are impossible, expanding the potential for renewable energy generation [8]. Floating platforms allow for rapid deployment and mobilization, reducing construction time and costs. They are used in bridge construction projects, embankment development, port development, and other facilities.

Modern developments are improving the functionality, safety, and efficiency of floating platforms. For example, composite materials [9], autonomous control systems, alternative energy sources, and digital technologies (e.g., digital twins for real-time platform optimization) are being used. Interest is also growing in “smart” platforms with sensors and data collection systems for monitoring condition and performance [10].

Climate change is a significant driver of the search for new technological solutions. Significant temperature fluctuations and aggressive environments necessitate the use of polymeric materials [11,12,13,14,15]. Engineers are considering new factors such as rising sea levels, increased frequency and intensity of storms, and changing temperature regimes. This requires the development of more resilient and adaptive designs.

Some of the challenges associated with the implementation of new solutions include the following:

- High initial capital investment, which may be inaccessible to small companies and requires the use of inexpensive yet effective materials and simple assembly conditions [16];
- The need to comply with strict safety and environmental regulations [17];
- The impact of adverse weather conditions on operations, necessitating the development of materials capable of operating effectively in low temperatures and aggressive environments [18,19,20].

Existing materials for floating platforms have a number of drawbacks that limit their use and operation. Sandwich panel materials using steel plates [21,22], which provide structural strength, are susceptible to rust, which over time leads to progressive deterioration of the structure. Reinforced concrete is also susceptible to degradation [23]: under the influence of seawater and rainwater, as well as in the presence of hydrogen sulfide, chlorine, and sulfur gases in the air, the reinforcement in the concrete mass corrodes, forming iron oxide products [24,25,26]. This leads to increased internal stresses and cracking of the concrete.

Concrete structures are more fragile than steel ones, which also imposes limitations on the development of floating platforms. Strong collisions or impacts can cause cracks in the hull of reinforced concrete platforms, increasing the risk of damage in rough seas.

Floating platforms are characterized by complex manufacturing and repair capabilities. The fabrication and operation of steel and reinforced concrete structures often involve complex processes (welding, waterproofing, formwork assembly, etc.), increasing time and cost. Some materials impose limitations on the shape and size of structures, which can be critical for specific applications.

An important limitation is the issue of durability and resource intensity. Structures made from traditional materials can have low durability and require significant lifecycle costs. This is exacerbated by exposure to extreme conditions. Concrete can be adversely affected by very low temperatures and aggressive substances. The solution to these shortcomings and limitations lies in the use of lightweight, durable materials capable of operating in low temperatures and aggressive environments. One such material is expanded polystyrene concrete, which provides buoyancy due to its low density and good thermal insulation properties [27,28,29]. An FEM model of foam concrete containing polystyrene spheres is proposed in [30]. This model allows for the prediction of strength and thermophysical properties based on the matrix properties and polystyrene concentration. However, studies [31,32,33] highlight the shortcomings of polystyrene concrete, manifested in changes in properties under environmental influences and exposure to fire or high temperatures. Polystyrene concrete is also susceptible to moisture; therefore, in an aqueous environment, polystyrene particles absorb water, increasing the weight of the structure and reducing its thermophysical and strength properties.

Materials such as steel and reinforced concrete are significant in weight, which increases the load on the structure, complicates transportation and operation, and requires more resources for maintenance.

To reduce the weight of structures, the use of vacuum (bubble) slabs in structures was considered in [34,35], offering several advantages, such as low dead weight, cost savings, and accelerated construction times. In [36], the authors studied slabs under four-point bending. The slabs contained 68 polystyrene balls of varying sizes (60 mm, 70 mm, and 90 mm), which were regularly distributed within the slabs. The authors examined a slab that was 3300 mm long, 1250 mm wide, and 250 mm thick. The slab was simply supported along its perimeter, with the distance between supports being 2850 mm. A weight reduction of approximately 6.4–21.6% was achieved by the authors, though a strength reduction ranging from 20% to 70% was observed. The authors note that the optimal ball size was 60 mm, which is half the slab height.

When using bubble slabs, it makes sense to remove concrete in the area of the neutral line of the span, since the neutral line is not deformed during bending. The slabs become lighter compared to traditional slabs, but their strength is reduced, and failure is brittle [19,27,37]. To improve ductility and deformability, the authors [38] used polymer reinforcement bars made of fiberglass. This design is lighter than traditional construction with steel reinforcement bars, but increases deflection after cracking because the fiberglass polymer has a lower modulus of elasticity than steel and undergoes greater deformation. Using bubbles with a diameter of 125 mm reduces the failure load even further and changes the failure mode of the structure. In general, the use of hollow-core slabs allows for a reduction in the volume of concrete by 25–50% [39,40]; however, the authors also note that a significant reduction in concrete leads to a decrease in the strength of the structure, which is confirmed by numerical and experimental studies [41,42,43,44,45]. The desire to remove concrete from the middle part and replace it with voids requires careful analysis, since the neutral line in concrete does not pass through the middle part of the slab [46]. It is known that the compressive and tensile strengths of concrete vary significantly. However, there is also a significant difference between the moduli of elasticity of concrete under compression and tension. This phenomenon is known as bimodularity. Considering the bimodularity of concrete, the neutral axis’s placement is a function of the reinforced concrete structure’s elastic and strength characteristics [47,48].

The limitations associated with the use of hollow-core slabs require careful analysis. An experimental analysis of slabs with polyethylene spheres under a static, centrally concentrated load was conducted in [49]. The experiment showed that solid traditional slabs outperform hollow-core slabs in terms of load-bearing capacity, although the latter are more efficient in terms of concrete savings. The authors reported that “the incorporation of polyethylene spheres not only decreases the self-weight of the slab but also enhances stress distribution and deformation performance”. The slabs exhibit predictable crack formation and satisfactory deformability. An FEM analysis of lightweight reinforced concrete structures with ellipsoid-shaped void formers for seismically active regions is presented in [50]. The authors obtained the eigenmodes and eigenfrequencies of vibrations, as well as the stiffness characteristics of the hollow-core slabs. In [51,52,53], an optimization of the numerical algorithm was performed based on the FEM analysis, which included minimizing computational costs and determining optimal block size parameters to balance material savings and structural performance. However, these studies do not provide a complete answer to the questions of void placement and the order of void formation.

This brief review has identified gaps in the existing research. It is clear that the use of polymeric materials as voids is a promising direction. At the same time, questions remain regarding the selection of void formation material properties, the order of their placement, the geometric characteristics of the voids, and their impact on load-bearing capacity and other thermophysical characteristics of the structure.

The study’s goal is to examine the influence of polymer-composite lightweight concrete composition on the mechanical and thermal properties that can be used in floating platforms. The research objectives can be summarized as follows:

1. Experimental investigation on the compressive behavior of lightweight concrete containing voids formed by hollow polypropylene spheres.
2. Development of an FE model of lightweight concrete with hollow spheres to determine the thermophysical properties and strength properties under compression.
3. Analysis of the stress–strain state of lightweight concrete with hollow spheres made of various polymer materials under compressive loads and comparison of numerical and experimental data.
4. Proposals for the development of a rational formulation and technological composition of lightweight concrete with hollow spheres.

## 2. Materials and Methods

### 2.1. Research Methodology

The methodology of this study is based on the authors’ previous studies [19,20,27], which initially involves experimental studies, followed by numerical modeling and comparison of results.

The experimental studies included the preparation of lightweight concrete with a control composition and compositions containing polypropylene spheres of varying diameters: 10 mm, 12 mm, 15 mm, 19.05 mm, and 20 mm. Six series of specimens were prepared for testing density, compressive strength, flexural strength, and thermal conductivity.

Numerical modeling was conducted in three stages. First, lightweight concrete was modeled as a two-phase solid with air voids. The resulting model formed the basis of the concrete matrix for the next stage—modeling lightweight concrete with polypropylene spheres of 10 mm, 12 mm, 15 mm, 19.05 mm, and 20 mm diameters (as in the experiment). Comparison with experimental results served to validate the numerical model. The third stage involved analyzing various polymeric materials by replacing polypropylene with polyamide 66 and polyester, and comparing the behavior of these polymeric materials. A comparison was made at this point to see how effective mixtures were with polymer spheres of varying diameters.

**Figure 1:**
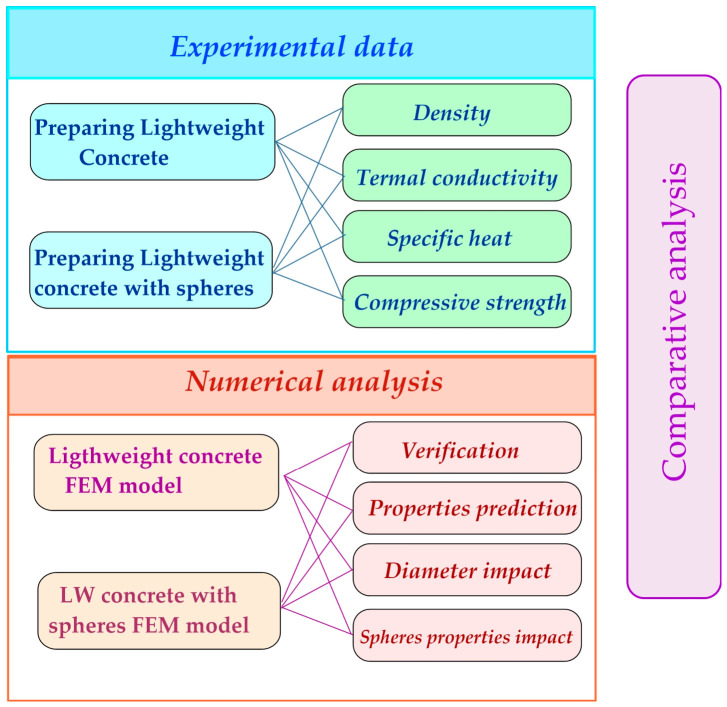
Research Diagram.

The diagram for this study is shown in Figure 1.

### 2.2. Materials

A summary of the raw material properties for lightweight concrete (LC) production is presented in Table 1. The material properties of Portland cement (PC) CEM I 42.5, Quartz sand (QZ) and Rospena (R) additive were used in [27].

**Figure 2:**
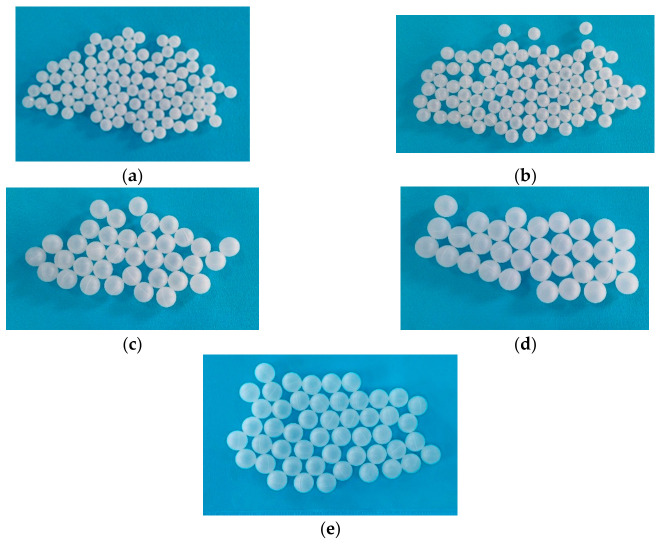
Polypropylene spheres: (**a**) 10 mm; (**b**) 12 mm; (**c**) 15 mm; (**d**) 19.05 mm; (**e**) 20 mm.

The appearance of polypropylene spheres of different diameters is shown in Figure 2.

Experimental lightweight concrete samples with polypropylene spheres of varying diameters were manufactured based on the formula given in Table 2.

The concrete mix for all types of compositions was prepared according to the following recipe: PC—390 kg/m^3^; QS—560 kg/m^3^; W—170 L/m^3^; Rospena—8 kg/m^3^. Initially, all raw materials required for the concrete mixture were measured in the specified proportions and introduced into a laboratory concrete mixer in the following order: Portland cement (PC) and quartz sand (QS), which were mixed for 60 s. The rotation speed of the laboratory mixer blades at this stage was 140 ± 5 rpm. Subsequently, mixing water and the Rospena admixture were added. The Rospena admixture was incorporated to enhance the rheological properties of the concrete mix, improve homogeneity and the uniform distribution of polypropylene spheres, and reduce the density of the concrete matrix through the formation of an additional fine-pore structure. Polypropylene spheres were poured into the ready-mixed concrete, and the entire composition was mixed for 60 s. The rotation speed of the blades of the laboratory mixer at the final stage of mixing the concrete mixture with polypropylene hollow spheres was 180 ± 5 rpm. Next, the ready-mixed concrete with polypropylene spheres was loaded into prepared metal prism molds measuring 40 × 40 × 160 mm. Molds containing fresh concrete mix were compacted on a laboratory vibrating table for 20 s at a frequency of 2900 ± 100 oscillations/min. Prismatic molds measuring 40 × 40 × 160 mm were chosen for the production of experimental composite samples in this experiment for reasons related to testing and practical aspects: the ability to more precisely control the material structure and the degree of distribution of polypropylene spheres within the cement–sand matrix; and the ability to conduct testing with simultaneous monitoring of destructive load and deformation. After 24 h, the lightweight concrete (LC) specimens were demolded and cured for an additional 27 days under laboratory conditions at a temperature of 20 ± 2 °C and a relative humidity of 60 ± 10%. The temperature of the freshly prepared concrete mix did not exceed 32 °C. Following 28 days of curing, the specimens were tested to determine density, compressive strength. In total, 18 prism specimens with dimensions of 40 × 40 × 160 mm were produced, with three specimens prepared for each mixture composition. For each experimental mixture composition (LC0PS, LC10PS, LC12PS, LC15PS, LC19PS, LC20PS) with polypropylene hollow spheres of different diameters, 3 samples were tested to ensure the reliability of the results and data comparison.

### 2.3. Experimental Methods

The density of the experimental lightweight concrete (LC) specimens containing polypropylene spheres was determined in accordance with the requirements specified in EN 12390-7:2019; Testing hardened concrete—Part 7: Density of Hardened Concrete [54]. Prior to testing, the specimens were dried to a constant mass, weighed, and their geometric dimensions were measured. The density values were then calculated using Equation (1) with an accuracy of 1 kg/m^3^.

(1) $$\rho =\frac{m}{V}\times \;1000$$

here *m* is the sample mass (g); *V* is the sample volume (cm^3^).

The density of each sample series was determined by calculating the arithmetic mean of the results obtained from three specimens. The measured density values are given in Table 3.

The compressive strengths were determined in accordance with the requirements of EN 196-1:2016; Methods of testing cement—Part 1: Determination of strength [55]. The choice of this testing method was determined by the size of the experimental composite samples, as well as the structure of the material, which is represented by a cement–sand matrix with randomly located inclusions in the form of polypropylene spheres. Prior to testing, the geometric dimensions of the PCLC specimens containing spheres with different diameters were measured. The compressive strength values were calculated using Equation (2), with an accuracy of 0.1 MPa. Mechanical tests were conducted using an S205 testing machine (Matest, Treviolo, Italy). During the compressive strength test, the specimens were subjected to loading at a constant rate of (0.6 ± 0.2) MPa/s.

(2) $$R=\alpha \frac{F}{A}$$

where *F* is the failure load (N), *A* is the cross-sectional area of the specimen (mm^2^), and α is a coefficient accounting for specimen dimensions (α = 0.95 for specimens with a side length of 100 mm).

The compressive strength of each specimen series was determined as the arithmetic mean of the test results obtained from six prism halves.

## 3. Results

### 3.1. Thermophysical Properties of Concrete with Hollow Spheres

**Figure 3:**
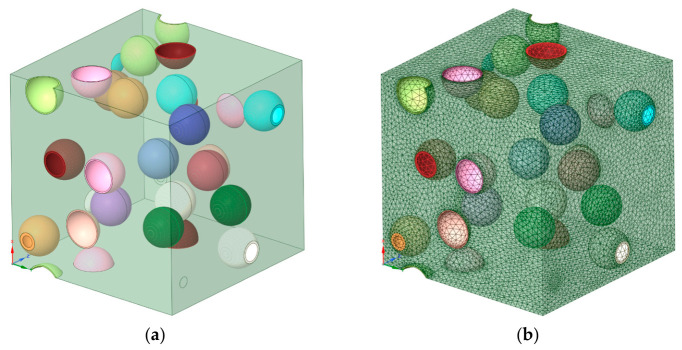
Representative volume of material with embedded hollow spheres: (**a**) geometric model; (**b**) finite element mesh for calculating thermophysical properties.

The thermophysical properties of polymer-composite materials play a crucial role in operational processes and influence the stress–strain state of the structure due to the heterogeneity of the temperature field. Therefore, the first step was to determine the thermal conductivity and specific heat capacity of lightweight concrete containing polymer spheres. A representative volume of the composite polymer material was generated in the Material Designer module, replicating the sphere content similar to that of the experimental samples. One version of this polymer-composite concrete is shown in Figure 3.

The base material is a concrete matrix with embedded hollow spheres. The properties of the LC and polymer spheres are presented in Table 4.

During the simulation, the variable parameters were the sphere material (polypropylene, polyethylene, or polyamide) and the sphere wall thickness. The volume fraction occupied by the spheres was assumed to be 8.7%, as in the experiment. Wall thickness varied from 0.1 mm to a solid sphere without an internal cavity.

The Material Designer module (ANSYS, version 2022 R1) allows you to model composite materials and determine their linear elastic and thermal properties using the Mori-Tanaka method. The following equations describe the calculation of orthotropic thermal conductivity. This calculation requires three loading cases, each creating a temperature gradient in the X, Y, and Z directions, respectively. For a material with orthotropic thermal conductivity, Fourier’s law defines the following relationship between the heat flux {*q*} and the temperature gradient ∇T:

(3) $$\left\{q\right\}=-\left[D\right]\;\;\nabla T$$

here [D] is the thermal conductivity matrix;

(4) $$\left[D\right]\;=\;\left(\begin{array}{ccc} K_{XX} & & \\ & K_{YY} & \\ & & K_{ZZ} \end{array}\right)$$

Here $K_{XX},\;K_{YY},\;K_{ZZ}$ is the thermal conductivity in directions X, Y, Z.

In the Material Designer module (ANSYS), a fixed temperature gradient is applied in the X direction, that is, there is a fixed value $\frac{\partial T}{\partial x}$ and $\frac{\partial T}{\partial y}=\frac{\partial T}{\partial z}=0$, then we obtain

(5) $$\left\{q\right\}=-K_{XX}\left(\begin{array}{l} \frac{\partial T}{\partial x}\\ \;\;0\\ \;\;0 \end{array}\right)$$

We seek the solution to Equation (5) in the form

(6) $$K_{XX}=-\frac{q_{X}}{\left(\frac{\partial T}{\partial x}\right)}$$

Here $q_{X}$ is the heat flux in direction X.

By integrating the heat flux across the boundary surface perpendicular to the X-axis, we easily obtain and thus obtain the thermal conductivity in the X-direction. Similarly, we obtain the thermal conductivity coefficients in the Y- and Z-directions.

**Figure 4:**
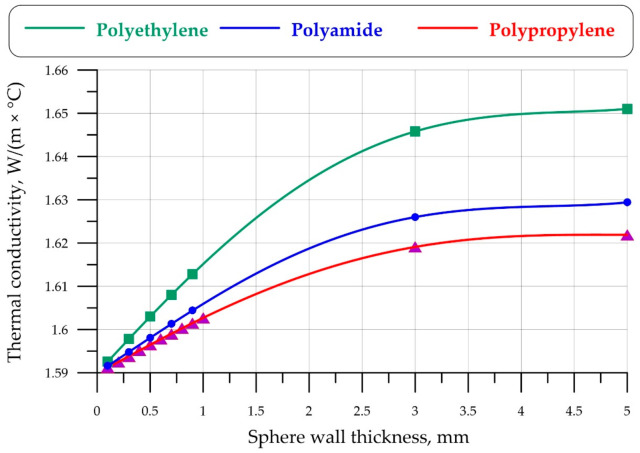
Dependence of the thermal conductivity coefficient of PCLC on the wall thickness of spheres for various polymeric materials: polyethylene, polypropylene, polyamide.

**Figure 5:**
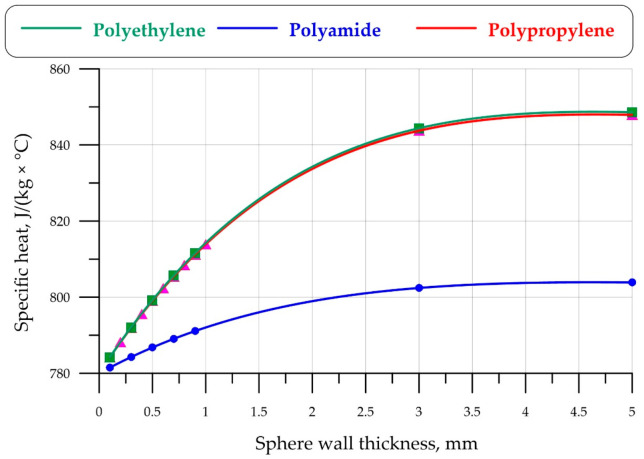
Specific heat of PCLC correlation on various sphere wall thicknesses for different polymer materials: polyethylene, polypropylene, polyamide.

The simulation results are presented in Figure 4 and Figure 5 as dependences of the thermal conductivity coefficient and specific heat capacity of PCLC on the sphere wall thickness for common polymeric materials.

The obtained dependences of thermal conductivity and heat capacity on wall thickness for different types of polymer materials can be scaled due to the linearity of the problem. In this case, a constant void fraction of 8.7% is considered, and only changes in wall thickness and the properties of the polymer materials are considered.

### 3.2. Construction and Verification of a Numerical Model of PCLC Specimens Under Compression and Bending

The numerical analysis of PCLC specimens under compression and bending was performed in the ANSYS Static Structural module. The constitutive model for lightweight concrete was based on “the Menetrey–Willam formulation, in which the yield surface describes a nonlinear stress–strain response under increasing loading”. Material softening was represented using an exponential function and initiated once the plastic potential function reached its peak value. The parameters of the Menetrey–Willam model were calibrated according to the recommendations provided in (Table 5) [27,32].

Numerical simulations of the PCLC specimens under compressive loading were conducted using the Static Structural module in ANSYS. Owing to the symmetry of the model, only one-quarter of the experimentally tested specimen was considered in the analysis. A parallelepiped measuring 20 mm × 20 mm × 160 mm occupies position in Cartesian space XYZ. Spheres with diameters of 10 mm (25% of the specimen height *h*), 12 mm (0.30 *h*), 15 mm (0.375 *h*), 19.05 mm (0.495 *h*), and 20 mm (0.5 *h*) were randomly generated within the specimen. The numerical analysis considered voids filled with air, voids without polymer, and voids filled with polymer spheres with a wall thickness that varied depending on the problem statement.

The boundary conditions of the problem were chosen to match the compression test conditions. A face displacement of *UX* = 8 mm was specified on the specimen surface at *z* = 160 mm. All nodes belonging to the *x* = 0 plane are fixed in the x-direction, *UX* = 0. All nodes belonging to the *z* = 0 plane are fixed in the z-direction, *UZ* = 0. All nodes belonging to the *y* = 0 plane are fixed in the y-direction, *UY* = 0.

**Figure 6:**
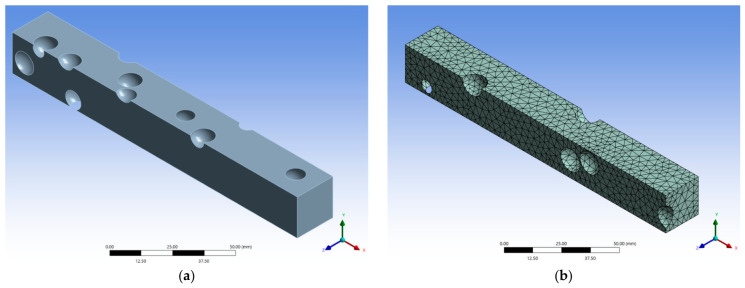
FEM elements of the PCLC model with 10 mm spheres: (**a**) geometry for 10 mm spheres; (**b**) finite element mesh for 12 mm spheres.

The finite element geometry and mesh are shown in Figure 6.

**Figure 7:**
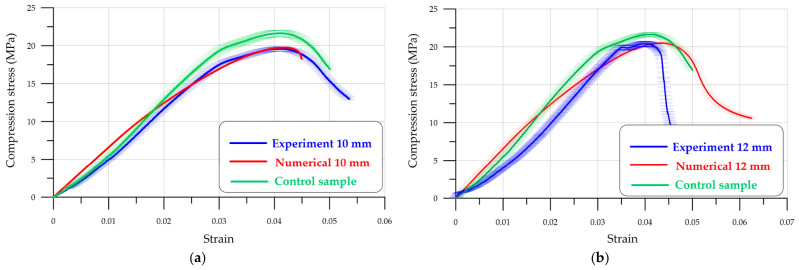
Comparison of the numerical analysis results for the PCLC model with experimental data (the blurred areas show the standard deviation of the experimental data): (**a**) 10 mm polypropylene spheres; (**b**) 12 mm polypropylene spheres.

The model was verified by comparing the results of numerical analysis and experimental data for compression specimens with 10 mm and 12 mm hollow spheres. The results of the data comparison are shown in Figure 7.

The results indicate that the numerical model adequately captures the compressive deformation behavior and closely reproduces the experimental load–deformation response in terms of both strength and deformation characteristics. For comparison, the figure shows the stress–strain curve of a control specimen without voids. The effect of the spheres is manifested in a decrease in compressive strength and a less pronounced failure pattern. It is also evident that the modulus of elasticity of lightweight concrete without voids is higher than that with spheres. Concrete without voids is stiffer than that with spheres, and the slope of the curve for the experimental specimen is greater than that of PCLC. This allows us to proceed to the main analysis of the stress–strain state and identify the failure characteristics of PCLC.

### 3.3. Analysis of the Stress–Strain State of PCLC Under Compression

**Figure 8:**
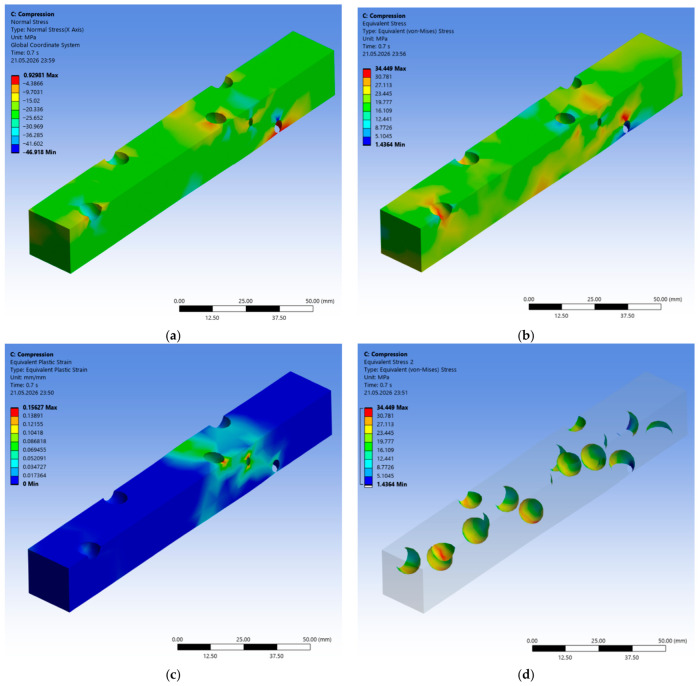
Stress–strain state of PCLC under compression at the moment of failure: (**a**) normal stresses $\sigma_{XX}$; (**b**) equivalent von Mises stresses $\sigma_{EQV}$; (**c**) equivalent plastic strain $\varepsilon_{EQV}$; (**d**) equivalent von Mises stresses in spheres.

The nature of the stress–strain state of PCLC under compression is determined by the presence of spherical voids and their relative positions. Spheres are stress concentrators and sources of crack formation, although of all geometric shapes, the sphere creates the least stress concentration. The stress and strain fields of a PCLC with 10 mm polypropylene spheres are shown in Figure 8.

**Figure 9:**
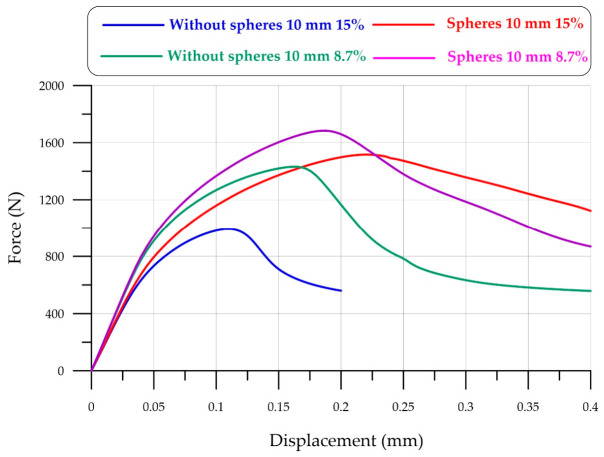
Effect of the proportion of hollow polypropylene spheres on the force-displacement curves.

Normal stresses (Figure 8a) and von Mises stresses (Figure 8b) indicate stress concentration in areas weakened by holes. Figure 8b shows areas (red) in which stresses exceed the yield strength, and failure occurs in these areas. Figure 8c shows areas of developed plastic deformation, which correspond to crack formation under compression. It can be seen that the active phase of the crack has already covered the entire specimen, and failure occurs in an inclined section relative to the X-axis. Figure 8d shows the equivalent stresses in the spheres. It can be seen that the spheres are subjected to significant deformation, but the stresses in the spheres do not exceed the strength characteristics of polypropylene. The spheres, due to their sufficient strength and rigidity, absorb part of the load, while the stress pattern, caused by a complex stress state in which hydrostatic stress plays a significant role, transfers some of the stress to the spheres. The effect of the spheres on the stress–strain curve is shown in Figure 9.

Figure 9 shows the force-displacement curves for concrete specimens with 10 mm voids without polymer (15% voids, blue curve, and 8.7% green curve) and with polypropylene spheres (15% sphere volume fraction, red curve, and 8.7% purple curve), obtained based on calculations in ANSYS. Figure 9 demonstrates that the presence of polymer spheres strengthens the specimen compared to the same number of voids but without polymer. However, a significant proportion of polymer spheres (15%) exhibits worse compressive properties than 8.7%. This is explained by the presence of a greater number of stress concentrators than with a smaller volume fraction of spheres.

**Figure 10:**
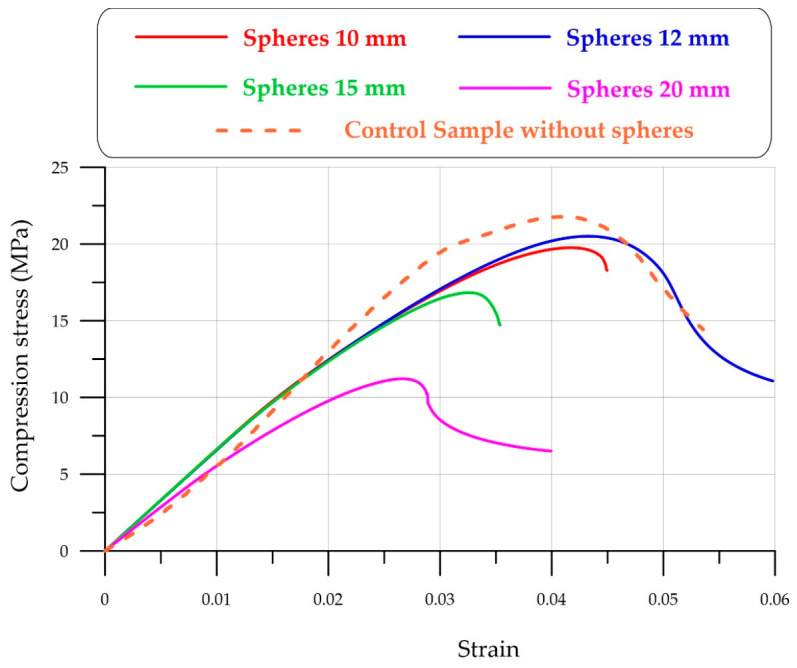
Stress–strain curves for polypropylene spheres with the same void fraction of 8.7%.

The effect of sphere diameter on specimen strength at the same void fraction is shown in Figure 10.

Figure 10 shows that the presence of 10 mm and 12 mm spheres (25% and 30% of the beam height) slightly reduces the specimen strength. The strength reduction is approximately 11%. For spheres with a diameter of 15 or 20 mm (37.5% and 50% of the beam height), the reduction is significant, which is explained by a significant reduction in the effective cross-section.

### 3.4. Effect of Polymer Sphere Material on the Strength Properties of the Specimen

Considering the favorable performance of spheres with diameters of 10 mm and 12 mm, the influence of different polymer materials on the stress–strain behavior of the specimens was further investigated using spheres with a diameter of 12 mm. Three types of polymer materials were considered as spheres: polypropylene, polyamide 66, and polyester. In the numerical model, the strength properties of the polymer materials were specified as a bilinear model with isotropic hardening. The material properties are summarized in Table 6.

**Figure 11:**
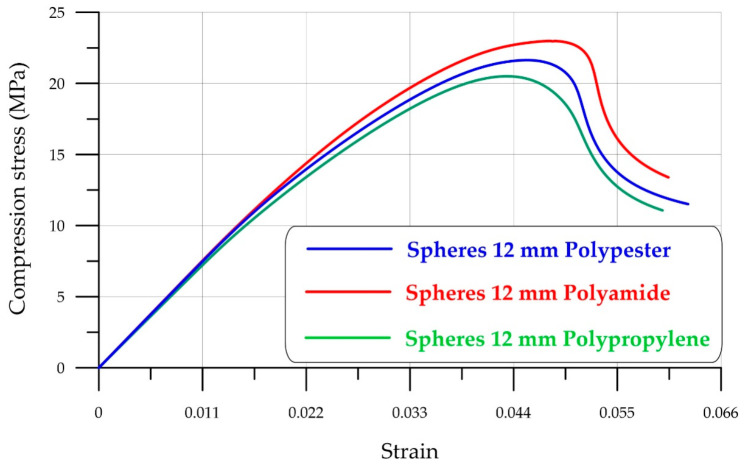
Stress–strain curves for Polypropylene, Polyamide 66, and Polyester.

The calculated stress–strain distributions for the three polymer types are presented in Figure 11.

**Figure 12:**
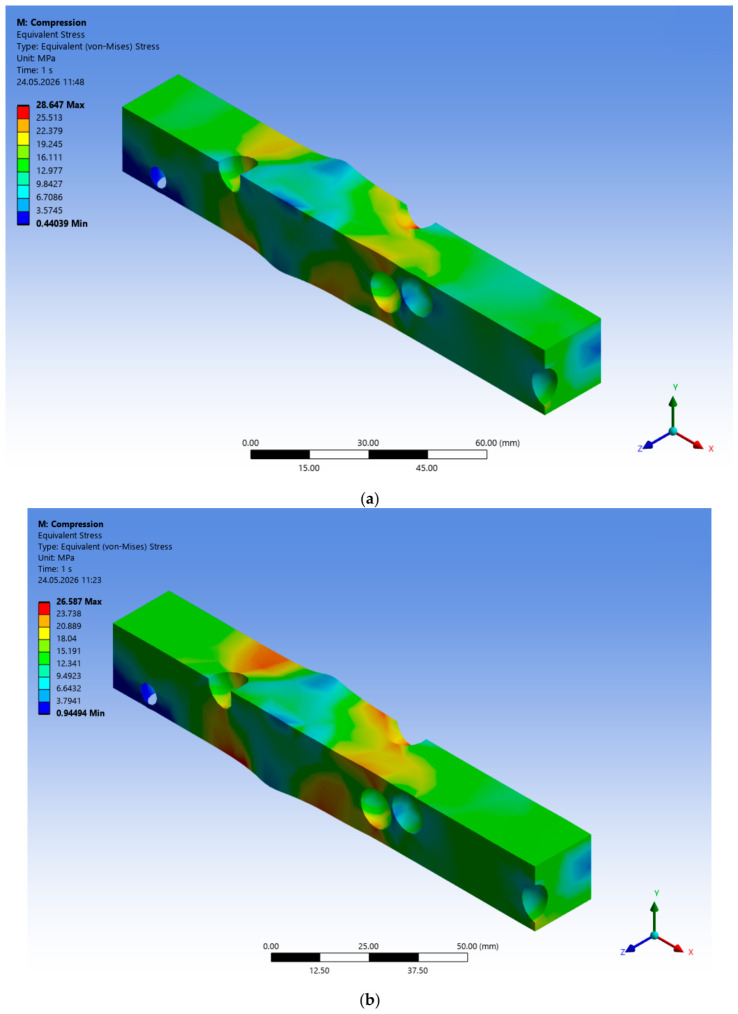
Equivalent von Mises stresses in specimens with 12 mm hollow spheres: (**a**) for polypropylene spheres; (**b**) for polyester spheres.

The stress–strain behavior is qualitatively similar for spheres of the same diameter. The differences are quantitative (Figure 11 and Figure 12).

**Figure 13:**
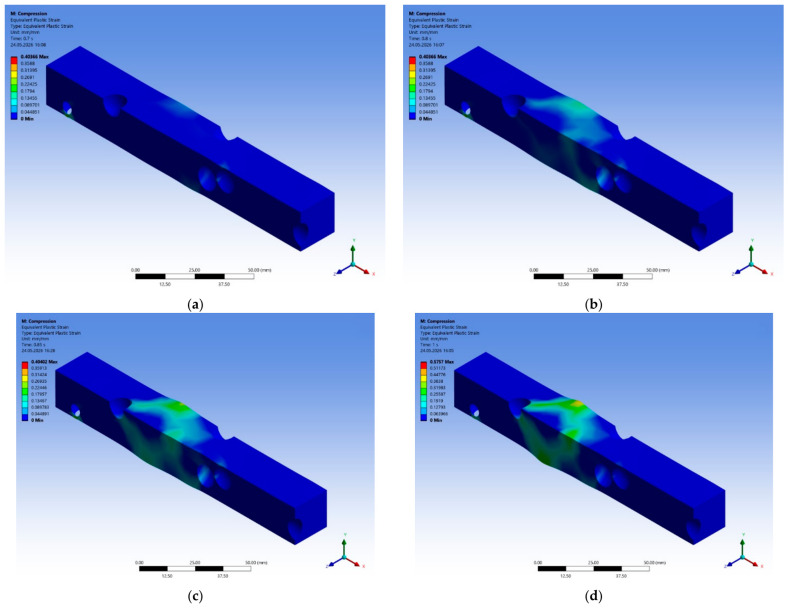
Sequential crack development in specimens with 12 mm polypropylene spheres: (**a**) 70% of the failure load; (**b**) 80% of the failure load; (**c**) 85% of the failure load;(**d**) 100% of the failure load.

Let us consider the sequential development of crack formation in a specimen with 12 mm polypropylene spheres. Figure 13 shows the development of equivalent plastic strain, which corresponds to crack propagation.

**Figure 14:**
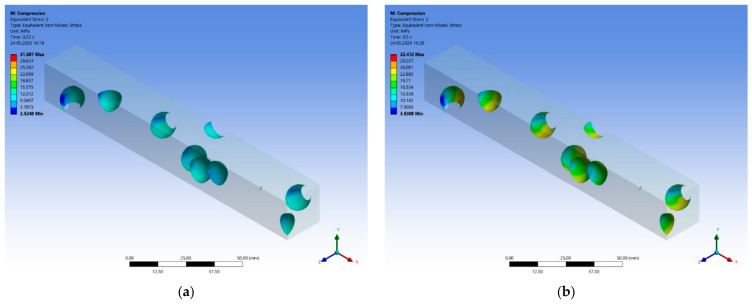

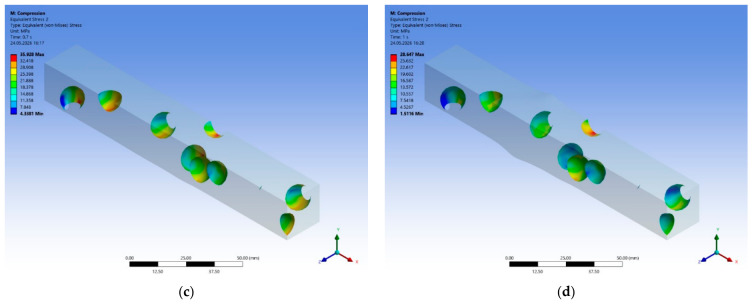
Sequential development of equivalent von Mises stresses in 12 mm polypropylene spheres: (**a**) at 25% of the failure load; (**b**) at 50% of the failure load; (**c**) 70% of the failure load; (**d**) 100% of the failure load.

**Figure 15:**
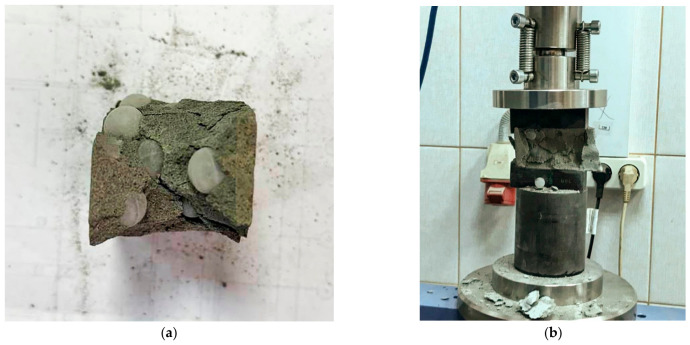
Photographs of specimens with 12 mm polypropylene spheres fractured under compression: (**a**) individual spheres are deformed; (**b**) specimen after fracture.

Comparing the stress fields in the spheres (Figure 13) and the crack development (Figure 14), it is evident that the polypropylene spheres are in a complex stress state and only at the failure load do the spheres begin to deform plastically. This indicates that the spheres have a sufficiently large safety factor, as confirmed by the experimental photographs (Figure 15).

**Figure 16:**
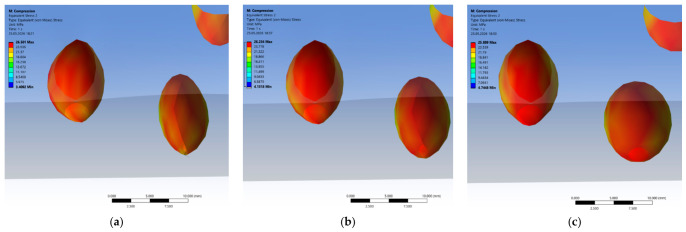
Deformation of 10 mm spheres at ultimate breaking load: (**a**) Polypropylene; (**b**) Polyamide 66; (**c**) Polyester.

The deformation of 10 mm diameter spheres for various polymer materials is shown in Figure 16.

Figure 16 shows that spheres made of polypropylene, which has the lowest modulus of elasticity, are most susceptible to deformation. Elasticity theory indicates that the deformation of a spherical shell under the action of external distributed pressure is proportional to the square of the shell’s radius [56]

(7) $$u_{r}∼\frac{R^{2}\rho g\left(1+\mu \right)}{E}$$

Here, *R* is the sphere’s radius, ρ is the shell density, *g* is the acceleration due to gravity, *E* is the modulus of elasticity, and μ is Poisson’s ratio.

From Equation (7), it is clear that the deformation of a sphere is proportional to the sphere’s radius and inversely proportional to the modulus of elasticity of the sphere’s material. From this perspective, it is logical to embed spheres of small radius and high modulus of elasticity in concrete. As Figure 16 shows, this is precisely how the spheres are deformed. That is, the deformation of the less rigid polypropylene is greater than that of polyamide 66 and polyester. This equation shows the general trend, in which the shell displacement is proportional to the sphere’s radius and inversely proportional to the elastic modulus. An accurate solution for a complex stress state depends on many factors. Modeling the behavior of an individual sphere and multiple spheres within a cement matrix at different stages of loading, up to and including failure, depends on a variety of factors, including the properties of the cement matrix, the geometry and properties of the spheres, the loading characteristics, and the boundary conditions.

## 4. Discussion

### 4.1. Thermophysical Characteristics of PCLC

This article presents a methodology and solves the important problem of predicting the thermophysical properties of PCLC depending on the geometry and properties of the cement matrix and polymer materials. Such problems play a significant role in the development of mixtures for 3D printing [57], thermal insulation architectural coatings [58], and bubble-deck technology [59]. In article [60], the authors conducted laboratory experiments, analyzed materials, and determined thermal characteristics to evaluate the effectiveness of using hollow balls from plastic bags as thermal insulation spheres inside Bubble Deck slabs. Plastic spheres with a diameter of 70 mm and a wall thickness of 10 mm were uniformly distributed within a 150 mm thick slab at a spacing of 50 mm. The findings of that study are in good agreement with the results reported in the present investigation. The authors [61] showed A loss of strength of almost half can be compensated for by a 30% improvement in thermal conductivity, which is effective in the conditions of the Persian Gulf countries, where summer temperatures can create unbearable living conditions. This article analyzes the thermophysical properties depending on the sphere diameter, wall thickness, and type of polymer material. This allows engineers to optimize the mixture composition to improve thermal properties without significant loss of strength.

### 4.2. Analysis of the Stress–Strain State of PCLC Specimens Under Compression

The strength properties of polymer-composite panels play a crucial role in their application in construction. Therefore, in selecting appropriate polymer materials and their geometric properties, it is essential to understand the stress–strain behavior of the polymer spheres within the specimen during the elastic, plastic, and failure stages of deformation.

Concrete slabs incorporating spherical void elements have gained considerable attention in recent years and have been applied in various construction projects [62]. Mohammed Ali et al. [40] discusses void formers used in concrete elements, which typically consist of hollow spheres made of high-density polyethylene or expanded polystyrene. The authors emphasized that the structural analysis of spherical hollow-core systems is more challenging due to their heterogeneous nature. An analysis of bending stresses in beams, conducted by the authors [63] using ABAQUS (version 6.12) software, did not allow for the analysis of the downward-sloping stress–strain curve. This is the primary focus of our study, since it is precisely at the stage of developed plastic deformation and at failure that polymer hollow spheres experience stresses close to the yield strength.

The failure mechanism of polymer composite specimens, detailed in this article, showed that crack formation initiates in the zone weakened by the spheres and propagates in the direction of maximum shear stress. This is illustrated in Figure 11, which shows the stages of crack development. In this case, the polymer hollow spheres absorb part of the potential deformation energy, compensating for the stresses in the concrete matrix. The deformation pattern of polymer spheres is shown in Figure 14 and is determined by the radius and elastic modulus of the polymer. In this regard, when designing polymer composite slabs, it is advisable to select spheres of small radius with a high elastic modulus. Similar results were obtained in [64], in which the authors described the nature of beam cracking during bending. Although the authors did not investigate the descending branch of the response curve, they demonstrated that concrete crushing occurred once the internal stresses in the compression zone exceeded the characteristic compressive strength.

Its worth noting that the research also contributes to a reduction in carbon footprint, as it allows the use of hollow spheres instead of cement. Article [65] reports a study showing that including lithium slag waste significantly increases tensile strain by 47.57%. Microstructural analysis revealed that lithium slag particles act as hydration nuclei and pore fillers, purifying the pore structure and optimizing properties. Flexural strength increased by 22.1%, and the carbon intensity index decreased by 8.18%.

Limitations of the model include insufficient consideration of interfacial behavior between the concrete matrix and the polymer sphere (assuming complete adhesion). For non-polar polymers, such as polypropylene, adhesion to the cement matrix is typically weak, which can affect stress transfer and crack propagation paths.

## 5. Conclusions

This study presents an experimental investigation and finite element (FEM) analysis of the thermophysical properties and compressive behavior of polymer-composite lightweight concrete containing polymer spheres embedded within the concrete matrix. The following key results are obtained:

- A lightweight concrete composition is proposed using hollow polymer spheres as the lightweight aggregate. The recommended ratio of raw components for the production of concrete mix is as follows: Portland cement—390 kg/m^3^; quartz sand—560 kg/m^3^; water—170 L/m^3^; Rospena additive—8 kg/m^3^. The preparation of lightweight concrete with polypropylene spheres of various diameters was carried out by mixing the concrete mix with hollow spheres at the following volume ratio: concrete mix—0.85 m^3^/m^3^; hollow polypropylene spheres—0.15 m^3^/m^3^. It is important to note that this composition is universal and, subject to the volume ratios, it is possible to use spheres of various diameters (10 mm, 12 mm, 15 mm, 19.05 mm, 20 mm). Incorporating hollow polymer spheres allows for concrete savings, which in the article amounted to 8.7% without significant loss of strength.
- An experimental study of PCLC was conducted to determine its thermophysical and strength properties. The observed reduction in PCLC strength compared to the control composition (*R_b_* = 21.4 MPa) without spheres is less than 11% for spheres with a diameter of 10 mm (*R_b_* = 19.1 MPa) and 12 mm (*R_b_* =18.2 MPa). For spheres with a larger diameter, the reduction in strength reaches 25% (*R_b_* = 16 MPa).
- A model for predicting the thermophysical characteristics of polymer composite concrete with polymer spheres is developed. The dependences of thermal conductivity and heat capacity on sphere wall thickness are obtained for various polymer materials. It is shown that, for a given sphere fraction of 8.7% of the concrete volume, the thermal conductivity improves by 12%. The obtained values allow for thermal calculations for polymer composite concrete structures subjected to thermal effects.
- A FEM was developed to analyze the stress–strain behavior of PCLC under compressive loading, considering material softening during failure. Stress–strain curves corresponding to different sphere sizes were obtained and compared with the experimental results. The model showed good agreement in predicting both strength and deformation characteristics.
- The failure mechanism of PCLC specimens was identified, showing that cracking initiates in the weakened zone of the spheres and propagates in the direction of maximum shear stress. The deformation pattern of the spheres is determined by the sphere radius and elastic modulus, allowing for the design of a mixture composition for various structures without significant loss of strength.

This study involves modeling a composite material consisting of a cement–sand matrix and lightweight aggregate in the form of hollow polypropylene spheres. The inclusion of polypropylene spheres in the concrete matrix allows for the creation of macropores of a specified size and a reduction in the density of the composite. Due to their regular geometric shape, polypropylene spheres make it possible to control the void content and density of the composite.

The value of this study is that, using a regular lightweight aggregate as an example, it provided a starting model for further refinement for more complex aggregates with different configurations and porosity patterns. Future research plans include developing this model for practical applications to predict the thermophysical properties of composites based on lightweight porous aggregates with different porosity patterns and irregular geometric shapes, such as expanded clay, agloporite, tuff, and pumice.

## Figures and Tables

**Table 1:** Properties of Raw Materials.

Material Title	Indicator	Actual Value
CEMENT I 42.5 N (PC) (CEMROS, Stary Oskol, Russia)	Initial Setting Time (h-min)	3–20
	End Setting Time (h-min)	4–40
	Normal Density (%)	28
	Compressive Strength after 28 Days (MPa)	51.3
	Bending Strength after 28 Days (MPa)	7.9
Quartz sand (QS) (Subsoil, Samara, Russia)	Bulk Density (kg/m^3^)	1346
	True Density (kg/m^3^)	2598
	Dust and clay content (%)	0.11
	Total residue on sieve no.:	
	5	0
	2.5	2.2
	1.25	5.2
	0.63	11.6
	0.315	44.4
	0.16	95.5
	Fineness modulus	1.59
Rospena (R) additive (Rospena, Moscow, Russia)	Density (kg/m^3^)	1100
	Proteins (%)	25
	Mineral salts (%)	4
	Mechanism of action	Air-entraining and plasticizing effect
Polypropylene (PS) spheres (Jinan, China)	Diameter (mm)	10, 12, 15, 19.05, 20
	Wall thickness (mm)	1
	Ball material	Polypropylene

**Table 2:** Ratio of components for producing experimental lightweight concrete samples with polypropylene spheres of varying diameters.

No.	Mixture Type	Concrete Mixture (m^3^/m^3^)	Polypropylene Spheres	
			(m^3^/m^3^)	Diameter (mm)
1	LC0PS	1	0	-
2	LC10PS	0.85	0.15	10
3	LC12PS	0.85	0.15	12
4	LC15PS	0.85	0.15	15
5	LC19PS	0.85	0.15	19
6	LC20PS	0.85	0.15	20

**Table 3:** Density values for concrete compositions obtained.

Composition Type	Density, kg/m^3^	Density Change (%)
LC	1945	0
PCLCd10	1809	−7.0
PCLCd12	1816	−6.6
PCLCd15	1840	−5.4
PCLCd19	1820	−6.4
PCLCd20	1855	−4.6

**Table 4:** Thermophysical Properties of Materials.

Material Type	Density, kg/m^3^	Isotropic Thermal Conductivity Coefficient, W/(m × °C)	Specific Heat at Constant Pressure, J/(kg × °C)
Lightweight concrete	1945	1.8	780
Polypropylene	950	0.2	2300
Polyethylene	960	0.4	2300
Polyamide	1400	0.25	1150

**Table 5:** Menetrey-Willam model parameters for a lightweight concrete matrix.

Title	Units	Value
Young’s modulus	MPa	700
Density	kg/m^3^	970
Poisson’s ratio		0.18
Uniaxial compressive strength	MPa	26.0
Uniaxial tensile strength	MPa	4.0
Biaxial compressive strength	MPa	50.0
Dilatancy angle	degree	10
Plastic strain at uniaxial compressive strength		0.01
Plastic strain at transition from power law to exponential softening		0.05
Relative stress at the start of nonlinear softening		0.33
Residual relative stress at transition from power law to exponential softening		0.85
Residual compressive relative stress		0.2
Mode 1 area specific fracture energy	N/m	100
Residual tensile relative stress		0.1

**Table 6:** Properties of polymeric materials.

Property	Polypropylene	Polyamide 66	Polyester
Young’s modulus, GPa	1.5	2.7	2.5
Poisson’s ratio	0.33	0.41	0.316
Bulk modulus, GPa	1.470	5.55	2.26
Shear modulus, GPa	0.564	1.06	0.95
Yield strength, MPa	25	65	40
Tangent modulus, MPa	0.1	0.1	0.1

## Data Availability

The original contributions presented in the study are included in the article, further inquiries can be directed to the corresponding author.

## Abbreviations

PCLC	Polymer-composite lightweight concrete
PP	Polypropylene
PA	Polyamide
PES	Polyester
PS	Polypropylene Spheres
PC	Portland cement
R	Rospena additive
LC	Lightweight concrete
